# Protective effects of cell permeable Tat-PIM2 protein on oxidative stress induced dopaminergic neuronal cell death

**DOI:** 10.1016/j.heliyon.2023.e15945

**Published:** 2023-04-29

**Authors:** Min Jea Shin, Won Sik Eum, Gi Soo Youn, Jung Hwan Park, Hyeon Ji Yeo, Eun Ji Yeo, Hyun Jung Kwon, Eun Jeong Sohn, Lee Re Lee, Na Yeon Kim, Su Yeon Kwon, Su Min Kim, Hyo Young Jung, Duk-Soo Kim, Sung-Woo Cho, Oh-Shin Kwon, Dae Won Kim, Soo Young Choi

**Affiliations:** a Department of Biomedical Science and Research Institute of Bioscience and Biotechnology, Hallym University, Chuncheon 24252, South Korea; b Department of Biochemistry and Molecular Biology, Research Institute of Oral Sciences, College of Dentistry, Gangneung-Wonju National University, Gangneung 25457, South Korea; c Department of Veterinary Medicine & Institute of Veterinary Science, Chungnam National University, Daejeon, 34134, South Korea; d Department of Anatomy and BK21 FOUR Project, College of Medicine, Soonchunhyang University, Cheonan-si 31538, South Korea; e Department of Biochemistry and Molecular Biology, University of Ulsan College of Medicine, Seoul 05505, South Korea; f School of Life Sciences, College of Natural Sciences Kyungpook National University, Taegu 41566, South Korea

**Keywords:** Tat-PIM2, Parkinson's disease, Oxidative stress, ROS, Apoptosis, Protein transduction, Protein therapy

## Abstract

**Background:**

Oxidative stress is considered as one of the main causes of Parkinson's disease (PD), however the exact etiology of PD is still unknown. Although it is known that Proviral Integration Moloney-2 (PIM2) promotes cell survival by its ability to inhibit formation of reactive oxygen species (ROS) in the brain, the precise functional role of PIM2 in PD has not been fully studied yet.

**Objective:**

We investigated the protective effect of PIM2 against apoptosis of dopaminergic neuronal cells caused by oxidative stress-induced ROS damage by using the cell permeable Tat-PIM2 fusion protein *in vitro* and *in vivo.*

**Methods:**

Transduction of Tat-PIM2 into SH-SY5Y cells and apoptotic signaling pathways were determined by Western blot analysis. Intracellular ROS production and DNA damage was confirmed by DCF-DA and TUNEL staining. Cell viability was determined by MTT assay. PD animal model was induced by 1-methyl-4-phenyl-1,2,3,6-tetrahydropyridine (MPTP) and protective effects were examined using immunohistochemistry.

**Results:**

Transduced Tat-PIM2 inhibited the apoptotic caspase signaling and reduced the production of ROS induced by 1-methyl-4-phenylpyridinium (MPP^+^) in SH-SY5Y cells. Furthermore, we confirmed that Tat-PIM2 transduced into the substantia nigra (SN) region through the blood-brain barrier and this protein protected the Tyrosine hydroxylase-positive cells by observation of immunohistostaining. Tat-PIM2 also regulated antioxidant biomolecules such as SOD1, catalase, 4-HNE, and 8-OHdG which reduce the formation of ROS in the MPTP-induced PD mouse model.

**Conclusion:**

These results indicated that Tat-PIM2 markedly inhibited the loss of dopaminergic neurons by reducing ROS damage, suggesting that Tat-PIM2 might be a suitable therapeutic agent for PD.

## Graphical abstract

**Figure:**
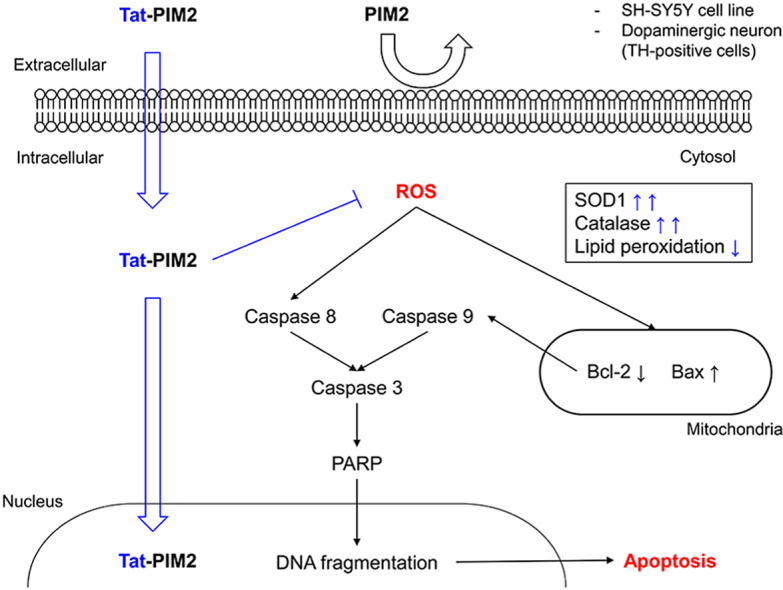

## 1 Introduction

Parkinson's disease (PD), one of degenerative disorder of the central nervous system, typically occurs in people aged over 60 years old and affects about 4% of the population aged over 80 years old [1] and main symptom has motor dysfunctions by loss of dopaminergic cell in the substantia nigra (SN) [2]. Although the precise mechanism of cell death is poorly understood, it implicates not only by accumulation of Lewy body but also by inflammation, and oxidative stress [[3], [4], [5], [6]]. In general, treatment of PD mostly involves the use of medications such as levodopa, MAO-B inhibitors or agonists of dopamine receptor, however those drugs lose effectiveness and shows adverse effects like involuntary muscle movements [5,[7], [8], [9]].

To investigate the relationship between PD and oxidative stress, 1-methyl-4-phenyl-1,2,3,6-tetrahydropyridine (MPTP) is usually used. MPTP-induced PD model has pathogenesis similar to human PD symptoms and shows dopaminergic neuronal cell death induced by oxidative stress-induced mitochondria dysfunction [3,10,11]. Several studies have shown increased oxidative stress in both PD patients and MPTP-induced PD animal models induced mitochondrial apoptotic pathway resulting the cell death by direct reactive oxygen species (ROS) action [[12], [13], [14]]. Moreover, the expression and regulation of caspase signaling have the critical role of distinguishing the progression of apoptosis caused by mitochondria dysfunction and ROS in PD [15,16].

Proviral Integration Moloney-2 (PIM2) protein, known as a serine/threonine-protein kinase family, is involved in the control of cell survival and apoptosis [[17], [18], [19]] and is highly expressed and regulated with external influences such as oxidative stress or inflammation in the brain and lymphoid cells [[20], [21], [22]]. Elevation of PIM2 reveals an anti-apoptotic function, which can be blocked by NF-κB inhibitor on multiple myeloma or lymphoma [[23], [24], [25]] and PIM2 knockdown mice model of gastric cancer augmented apoptosis and diminished the proliferation of cells by high levels of ROS induced by PIM2 silence [26].

Protein transduction domains (PTDs) facilitate the transduction of proteins into cells as well as various tissues including the brain [[27], [28], [29]] and we previously demonstrated that several PTD fusion proteins penetrated into cells and brain tissues passing through the BBB and showed inhibition of apoptosis [[30], [31], [32], [33]]. Therefore, cell permeable Tat-PIM2 was prepared and investigated the protective effects of this fusion protein against MPP^+^- and MPTP-induced oxidative damage in dopaminergic neurons using *in vitro* and *in vivo* PD models.

## 2 Materials and methods

### 2.1 Chemicals and cell culture

Fetal bovine serum (FBS) and antibiotics (streptomycin and penicillin) were obtained from Gibco BRL (Grand Island, NY, USA). Eagle's Minimum Essential Media (EMEM) was obtained from Lonza/BioWhittaker (Walkersville, MD, USA). Dichlorofluorescein diacetate (DCF-DA), 1-methyl-4-phenylpyridinium (MPP^+^) and 1-methyl-4-phenyl-1,2,3,6-tetrahydro pyridine (MPTP) were purchased from Sigma-Aldrich (St. Louis, MO, USA). All antibodies information used for Western blotting is in Table 1. All other reagents used were of guaranteed or analytical grade. Tat-PIM2 protein purified as previously described [34].

**Table 1:** Information of the primary antibodies used for Western blotting.

Name	Dilution	Source	Catalog number
6X Histidine	1:5,000	Abcam	ab9108
Bcl-2	1:1,000	Abcam	ab59348
Bax	1:1,000	Cell Signaling Technology	#2772
Csapase-3	1:1,000	Cell Signaling Technology	#9662
Cleaved Caspase-3	1:1,000	Cell Signaling Technology	#9664
Csapase-8	1:1,000	Cell Signaling Technology	#4790
Cleaved Caspase-8	1:1,000	Cell Signaling Technology	#9496
Csapase-9	1:1,000	Cell Signaling Technology	#9504
Cleaved Caspase-9	1:1,000	Cell Signaling Technology	#9509
PARP	1:1,000	Cell Signaling Technology	#9532
Cleaved PARP	1:1,000	Cell Signaling Technology	#9544
SOD	1:1,000	Cell Signaling Technology	#37385
Catalase	1:1,000	Cell Signaling Technology	#14097
4-HNE	1:1,000	Abcam	ab48506
8-OHdG	1:1,000	LifeSpan BioSciences	LS-C68291
β-Actin	1:10,000	Cell Signaling Technology	#4967

SH-SY5Y, human neuroblastoma cells, was cultured using EMEM with 15% FBS, 2 mM l-glutamine, and 100 μg/ml gentamicin in an incubator (37 °C, 5% CO_2_).

### 2.2 Analysis of Tat-PIM2's permeability in SH-SY5Y cells

To assess permeation of Tat-PIM2, it treated in SH-SY5Y cells by concentration-dependent manner (0.5–3 μM) for 1 h or time-dependent manner (10–60 min) with a dose of 3 μM, respectively. Then, Tat-PIM2 in intracellular was detected by Western blotting with anti-histidine antibody. To avoid interference of cell membrane surface binding, the cells exposed to Tat-PIM2 were washed with trypsin-EDTA (Gibco Grand Island, NY, USA) and PBS. Furthermore, to determine the stability of permeated Tat-PIM2 in SH-SY5Y, the cells were further incubated (1–48 h) and the levels were determined by Western blotting.

### 2.3 Western blotting

SH-SY5Y cells and mouse brain tissue samples were lysed in PRO-PREP™ Protein Extraction Solution (iNtRON Biotechnology, Gyeonggi-do, Korea), and centrifuged for 30 min at 4 °C in 15,000 rpm. The supernatant was boiled with sample buffer (EBA-1052, Elpis Biotech., Daejeon, Korea) for 5 min. The samples were loaded by 12% SDS-PAGE and were then transferred to a nitrocellulose membrane. The membranes were incubated with 5% skim milk in TBST for 1 h and were incubated with primary antibody overnight at 4 °C. After that these were incubated with HRP-conjugated antibody for 2 h. The protein bands were utilized chemiluminescent reagents (Amersham, Franklin Lakes, NJ, USA), and detected using Chemidac system (BioRad, USA).

### 2.4 Immunofluorescence staining

In previous studies, the distribution of permeable protein were used immunofluorescence staining and evaluated by confocal laser scanning microscope (FV-300, Olympus, Tokyo, Japan) [[35], [36], [37]]. Briefly, SH-SY5Y cells, which Tat-PIM2 (3 μM) treated for 1 h, were fixed by 4% paraformaldehyde. The fixed cells were treated with histidine primary antibody (1:2,000) for 1 h, and Alexa Fluor 488 conjugated secondary antibody (1:15,000, Invitrogen, Carlsbad, CA, USA) for 1 h at 37 °C. Nuclei were stained with 4′6-Diamidino-2-phenylindole (DAPI) for 2 min at room temperature.

### 2.5 Cell viability assay

Cell viability assay for cytotoxicity and effect of Tat-PIM2 was performed using MTT assay [32,38]. In the assay of cytotoxicity, Tat-peptide, PIM2, and Tat-PIM2 were treated with various concentrations (0–3 μM) for 24 h in SH-SY5Y cells. To evaluate the cell viability of Tat-PIM2, the proteins (0–3 μM) were pretreated for 1 h and treated 5 mM MPP^+^ for 12 h in SH-SY5Y cells. Both assays were detected by a Labsystems Multiskan MCC/340 plate reader (Farnborough, UK).

### 2.6 Measurement of ROS production

To measure the ROS production, the cells were pretreated with Tat-PIM2 for 1 h, which were incubated with MPP^+^ (5 mM) for 6 h. Then, the cells were stained with 5 μM 2′,7′-Dichlorofluorescein diacetate (DCF-DA) solution for 30 min. Images and intensity of the fluorescence were obtained using a fluorescence microscope (Nikon eclipse 80i, Tokyo, Japan) and Fluoroskan ELISA plate reader (Labsystems Oy, Helsinki, Finland), respectively [35,39].

### 2.7 TUNEL assay

Terminal deoxynucleotidyl transferase dUTP nick end labeling (TUNEL) was used to analyze the DNA damage according to the manufacturer's instructions [32,40]. The cells were pretreated with Tat-PIM2 for 1 h, which were incubated with MPP^+^ (5 mM) for 10 h. Then, TUNEL staining was performed. The stained cells with TUNEL were observed under a fluorescence microscope. The number of TUNEL positive cells did counting from the microscopy imaging data.

### 2.8 PD animal model

C57BL/6 mice (Male, 6-week-old) were purchased from the Hallym University Experimental Animal Center, managed on constant surroundings at 23 °C temperature and 60% relative humidity with 12 h light-dark cycle. All experimental procedures involving animals and their care conformed to the Guide for the Care and Use of Laboratory Animals of the National Veterinary Research and Quarantine Service of Korea and were approved by the Institutional Animal Care and Use Committee of Soonchunhyang University [SCH16-0052-01].

To identify the distribution of Tat-PIM2 in mice brain, the mice (n = 7/each group) were treated Tat-PIM2 (2 mg/kg) by intraperitoneally (i.p.) injected. The valid concentration of Tat-PIM2 (2 mg/kg) was decided following our previous studies [33,35]. After 12 h, the mice brain was sectioned and stained using an immunohistochemical method as previously described [33,35]. In order to analyze the effects of Tat-PIM2 on PD animal model, Tat-PIM2 (2 mg/kg) was injected into the mice (n = 7/each group) by i.p. method. After one day, MPTP (20 mg/kg) was injected into mice 4 times each at 2 h intervals, respectively [38]. A week later, the mice were sacrificed and performed immunohistochemistry with anti-tyrosine hydroxylase (TH), TH + histidine, TH + cresyl violet (CV). TH- and TH + CV-positive cells were displayed via microscopic counts as described previously [32,36].

### 2.9 Statistical analysis

All statistical data were used Graphpad Prism 8.0 software (Graphpad Software, San Diego, CA, USA). Values are shown as means ± standard error of the mean (S.E.M.) from three or more independent experiments. Statistical comparisons between the different treatments were performed using one-way analysis of variance (ANOVA) with Bonferroni's post-hoc test. A p-value <0.05 was considered to be statistically significant.

## 3 Results

### 3.1 Preparation, cellular uptake, and intracellular localization of permeable Tat-PIM2

**Fig. 1:**
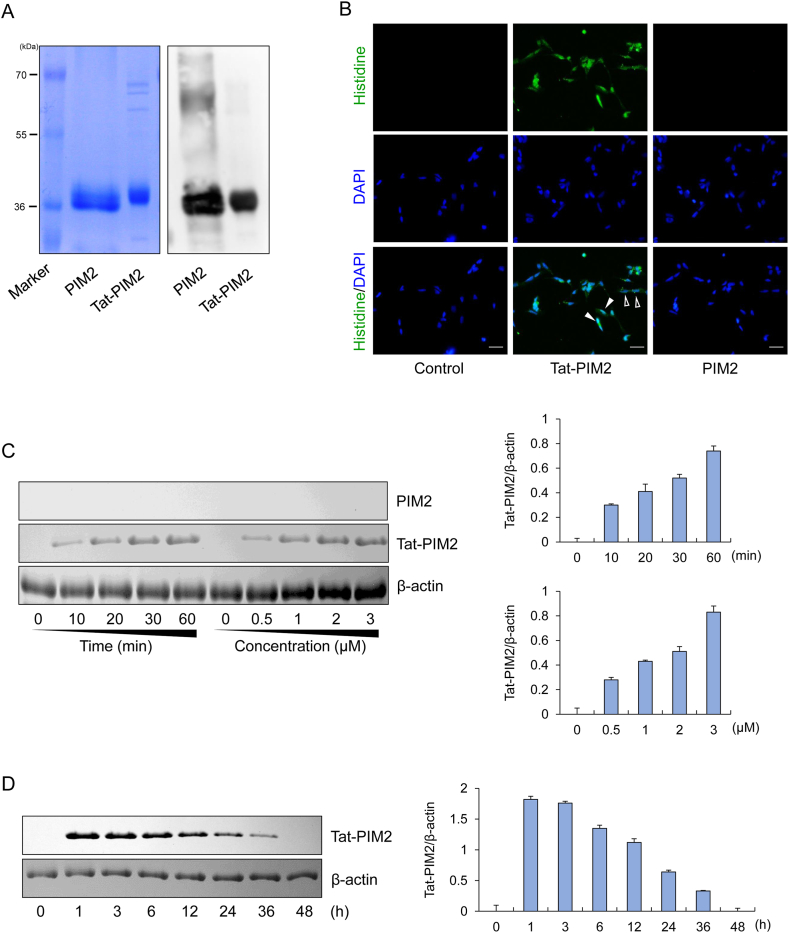
Preparation, cellular uptake, and intracellular localization of permeable Tat-PIM2. Purified Tat-PIM2 and PIM2 were identified by 12% SDS-PAGE and were confirmed by Western blot analysis using an anti-histidine antibody (A). SH-SY5Y cell culture media were treated with Tat-PIM2 or PIM2 (3 μM) for 1 h. Cellular localization of transduced Tat-PIM2 proteins was confirmed by fluorescence microscopy (B). Scale bar = 20 μm. SH-SY5Y cell culture media were treated with Tat-PIM2 and PIM2 at different concentrations (0.5–3 μM) for 1 h and were treated with Tat-PIM2 and PIM2 (3 μM) for different time periods (10–60 min) (C). Intracellular stability of transduced Tat-PIM2 (D). The cell culture media were incubated for 48 h after transduction of Tat-PIM2 for 1 h. Then, transduction of Tat-PIM2 was measured by Western blotting and the intensity of the bands was measured by a densitometer. The non-adjusted full images for immunoblots are shwon in Fig. S1.

In a previous study, we constructed recombinant PIM2 protein using Tat-peptide [34]. The purified Tat-PIM2 and control PIM2 were identified using SDS-PAGE and Western blotting (Fig. 1A). To confirm the cellular uptake and intracellular localization of Tat-PIM2, immunofluorescence using anti-histidine antibody and DAPI was used. Cell-permeable Tat-PIM2 was located in cytosol and nucleus of the SH-SY5Y cells half-and-half. On the other hand, PIM2, as a negative control, was not identified in the cells (Fig. 1B). Based on Western blotting, the permeability of Tat-PIM2 was affected by concentration and time (Fig. 1C). We also measured the stability of cell-permeable Tat-PIM2 for 48 h. Tat-PIM2 which had penetrated cells was stable for 36 h (Fig. 1D).

### 3.2 Effect of Tat-PIM2 on oxidative stress-induced cell death and ROS production

**Fig. 2:**
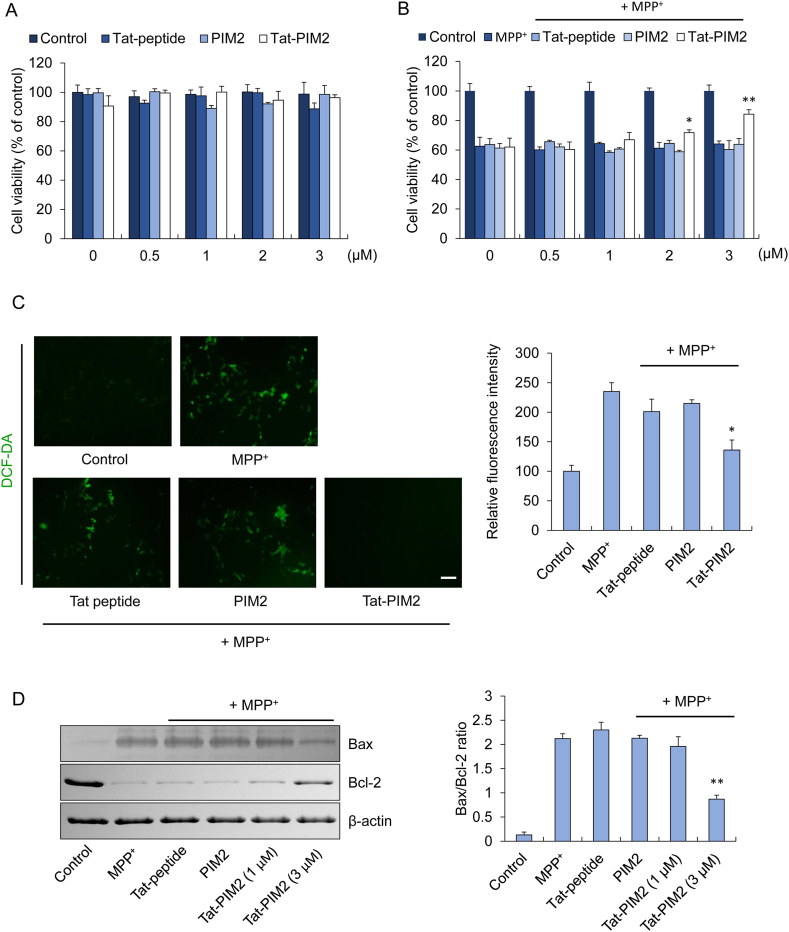
Effect of Tat-PIM2 on oxidative stress-induced cell death and ROS production. To measure the cytotoxicity, Tat-peptide, PIM2, and Tat-PIM2 were treated with various concentrations (0–3 μM) for 24 h in SH-SY5Y cells (A). Pretreatment of SH-SY5Y cells with Tat-PIM2 (0–3 μM) for 1 h and treatment with 5 mM MPP^+^ for 12 h. Then, cell viability was assessed by MTT assay (B). SH-SY5Y cells were treated with Tat-PIM2 (3 μM) for 1 h before treatment with 5 mM of MPP^+^ for 6 h. Then, intracellular ROS levels were determined by DCF-DA staining. Fluorescence intensity was quantified using an ELISA plate reader (C). Scale bar = 50 μm. Effects of Tat-PIM2 on MPP^+^-induced apoptotic mitochondrial protein expression in SH-SY5Y cells. The cells were treated with Tat-PIM2 (3 μM) for 1 h before being treated with MPP^+^ (5 mM). Bcl-2 and Bax expression levels were analyzed by Western blotting (D). Band intensity was measured by densitometer. *P < 0.05 and **P < 0.01 compared with MPP ^+^ treated cells. The non-adjusted full images for immunoblots are shwon in Fig. S2.

Prior to evaluating its efficacy, we analyzed the cytotoxicity of Tat-PIM2 on SH-SY5Y cells using an MTT assay, and our results did not show significant cytotoxic properties (Fig. 2A). MPP^+^ elicits oxidative stress that produces ROS and triggers cell death [35]. As shown in Fig. 2B, Tat-PIM2 exhibited a protective effect against MPP^+^-induced cell death. In cells treated with Tat-PIM2, viability increased from 62% to 84.3%. By contrast, PIM2 and Tat-peptide did not improve cell viability in MPP^+^-induced cell death (Fig. 2B).

We also assessed whether Tat-PIM2 could moderate cell damage due to ROS production caused by MPP^+^. Intracellular ROS production was increased in the MPP^+^ treated group, whereas ROS production was conspicuously reduced in the co-treated Tat-PIM2 group (Fig. 2C). Furthermore, mitochondrial proteins, such as, Bax and Bcl-2, were measured to confirm the protective effects of Tat-PIM2 in mitochondria-mediated cell damage caused by ROS production [26]. Tat-PIM2 showed a protective effect on the cells through decreased Bax level and increased Bcl-2 level as compared with the MPP^+^ treated group (Fig. 2D).

### 3.3 The inhibition effect of Tat-PIM2 on the apoptosis pathway

MPP^+^ generates ROS production which triggers the activation of apoptosis signaling pathways [13]. ROS-induced caspase pathway is particularly important in programmed cell death, including Caspase 8, caused by ASK1 activation, and Caspase 9, which results from mitochondrial damage [15]. Caspase 8 and 9 simultaneously enhance Caspase 3 activity and the expression of PARP, which causes DNA fragmentation in the apoptosis pathway [15,33].

**Fig. 3:**
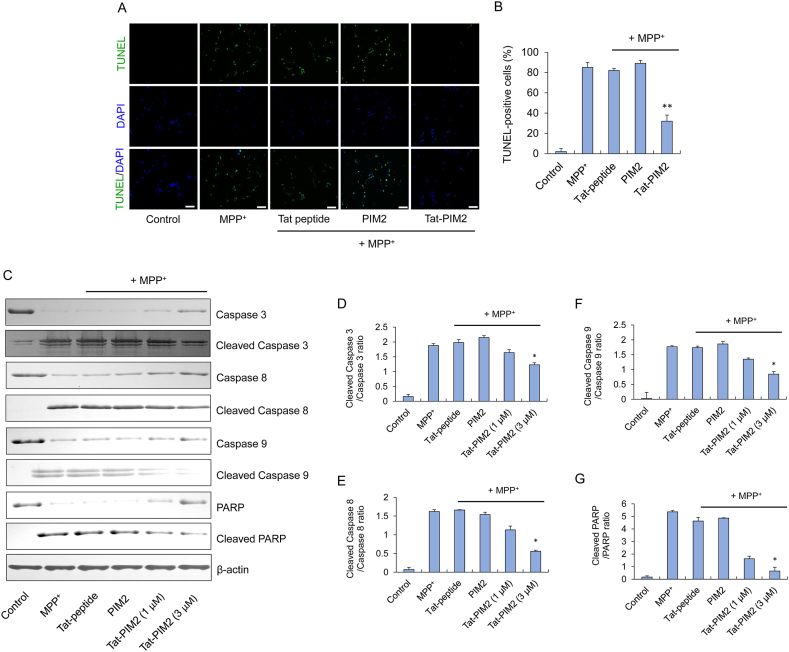
The inhibition effect of Tat-PIM2 on the apoptosis pathway. Effects of Tat-PIM2 against MPP^+^-induced DNA fragmentation. SH-SY5Y cells were treated with Tat-PIM2 (3 μM) for 1 h before treatment with 5 mM of MPP^+^ for 10 h. Then, DNA fragmentation was determined by TUNEL staining (A). TUNEL-positive cells were counted by phase-contrast microscopy ( × 200 magnification) for the cells (B). Scale bar = 50 μm. Effects of Tat-PIM2 on MPP^+^-induced apoptotic protein expression in SH-SY5Y cells. The cells were treated with Tat-PIM2 (3 μM) for 1 h before being treated with MPP^+^ (5 mM). Caspase-3, Cleaved Caspase-3, Caspase-8, Cleaved Caspase-8, Caspase-9, Cleaved Caspase-9, PARP, and Cleaved PARP expression levels were analyzed by Western blotting (C). Band intensity was measured by densitometer (D–G). *P < 0.05 and **P < 0.01 compared with MPP ^+^ treated cells. The non-adjusted full images for immunoblots are shwon in Fig. S3.

We thus confirmed the effects of Tat-PIM2 in the apoptosis pathway by assessing DNA fragmentation and caspase signaling. As shown in Fig. 3A, MPP^+^-induced DNA fragmentation by green fluorescence measured by TUNEL assay was not detected in the PIM2 and the Tat-peptide groups. On the contrary, decreasing fluorescent signal in apoptotic cells in the Tat-PIM2 group highlighted that DNA fragmentation was strongly diminished. To determine the role of Tat-PIM2 in MPP^+^-induced apoptosis pathway, cells were treated with Tat-PIM2 and 5 mM MPP^+^ for 8 h treated and the expression levels of apoptotic proteins was measured by Western blot analysis. In the 3 μM Tat-PIM2 treated group, expression levels of PARP, Caspases 3, 8, and 9 were restored, whereas in the group treated with PIM2 and Tat-peptide not protective effects were observed (Fig. 3C–G). In SH-SY5Y cells, MPP^+^ can be stimulated by the activation of apoptosis, whereas permeable Tat-PIM2 inhibited the apoptosis pathway thereby inhibiting caspase signaling.

### 3.4 Protective effect of Tat-PIM2 in Parkinson's disease animal model

**Fig. 4:**
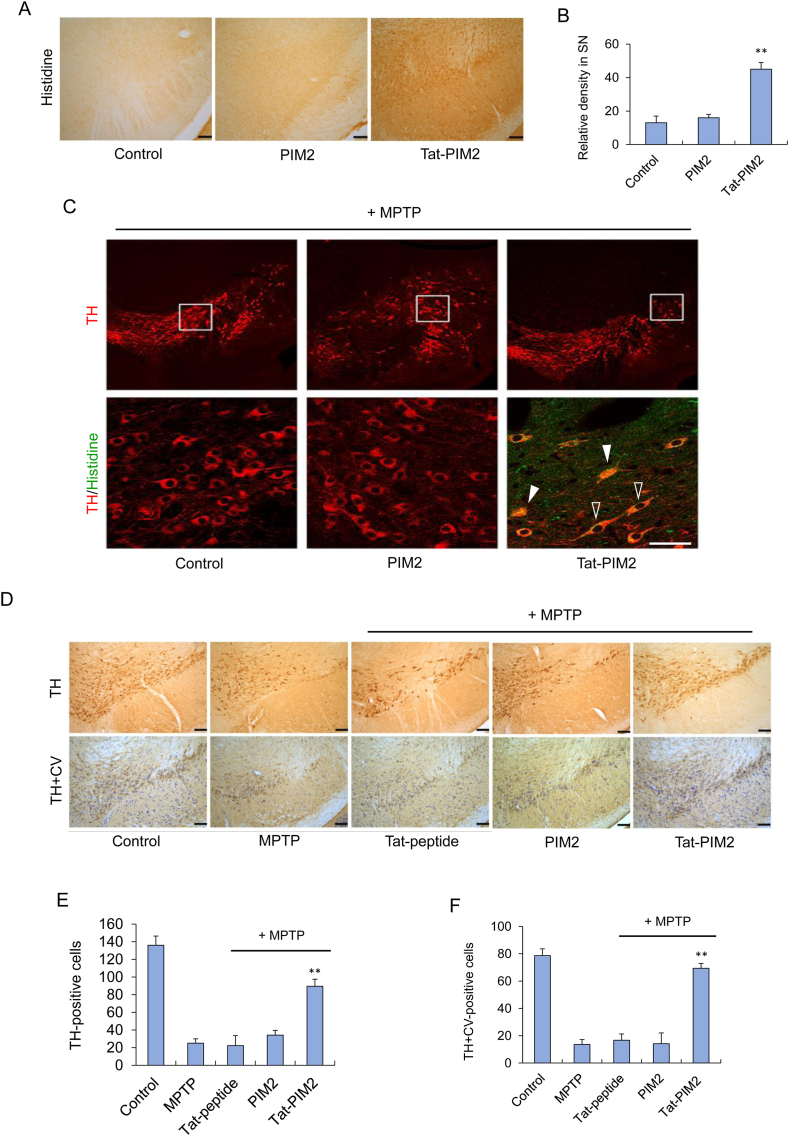
Transduced Tat-PIM2 inhibits dopaminergic neuronal cell death in PD animal model. Transduction of Tat-PIM2 into the SN. Tat-PIM2 (2 mg/kg) was injected i.p. into mice, followed by collecting the brains 12 h later. Brain tissues were immunostained with an anti-histidine antibody (A). Relative density was measured by densitometer (B). Localization of transduced Tat-PIM2 proteins was confirmed by fluorescence microscopy. Mice were treated with single injections of Tat-PIM2 (2 mg/kg) proteins and killed after 12 h. Tat-PIM2 was analyzed by immunohistochemistry using anti-Histidine and tyrosine hydroxylase (TH) (C). Protective effects of transduced Tat-PIM2 on PD animal model. Tat-PIM2 (2 mg/kg) was injected i.p. into mice, followed by collecting the brains for 1 week. Brain sections showing tyrosine hydroxylase (TH) immunoreactivity and double staining with cresyl violet (CV) and TH immunoreactivity (D). Scale bars = 100 μm. The number of TH-positive neurons. Quantification of the number of positive dopaminergic neurons in 250 × 250 μm^2^ is shown in the graph (E and F). *P < 0.05 and **P < 0.01, the statistically significant difference between MPTP and other groups. (For interpretation of the references to colour in this figure legend, the reader is referred to the Web version of this article.)

To confirm whether Tat-PIM2 could reach mice brain by passing through the BBB, Tat-PIM2 (2 mg/kg) was injected intraperitoneally and brain tissues were collected after 12 h. The distribution of Tat-PIM2 was measured by immunohistochemistry using a Histidine antibody. It was found that Tat-PIM2 successfully passed the BBB and was widely spread in the SN region of the mice brain (Fig. 4A and B). To determine the localization of Tat-PIM2, we performed immunocytochemistry in the SN region using anti-TH, a marker of dopaminergic neuron, and anti-His. As shown in Fig. 4C, Tat-PIM2 was significantly localized on dopaminergic neurons. Furthermore, Tat-PIM2 was situated in cytosol (full arrows) and nuclei (empty arrows) of TH-positive cells.

We also assessed whether Tat-PIM2 could reduce the death of dopaminergic neurons in MPTP-induced PD model using a TH antibody and cresyl violet perchlorate (Fig. 4D). It was found that Tat-PIM2 exhibited a protective activity on dopaminergic neurons by increasing the number of TH- and TH + CV positive cells in the SN, but no protective effects were observed in the presence of PIM2 or Tat-peptide (Fig. 4E and F).

### 3.5 The role of Tat-PIM2 on signaling mechanisms in the MPTP animal model

**Fig. 5:**
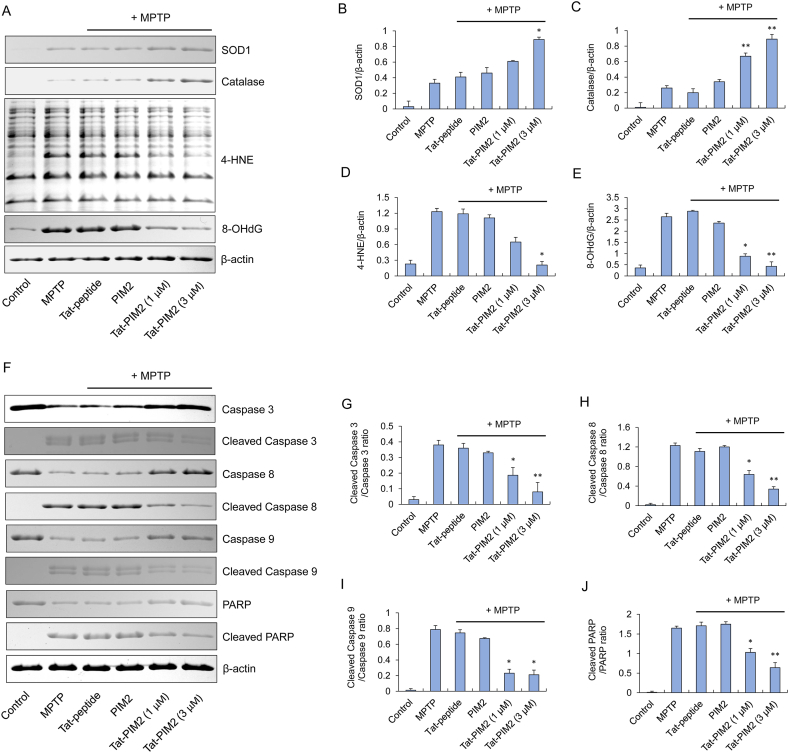
The role of Tat-PIM2 on signaling mechanisms in the MPTP animal model. Tat-PIM2 (2 mg/kg) was injected i.p. into mice, followed by collecting the brains for 1 week. Intracellular antioxidant-related protein expression levels using Western blot analysis by anti-SOD1, Catalase, 4-HNE, and 8-OHdG (A). Band intensity was measured by densitometer (B–E). The apoptotic protein expression levels using Western blotting analysis by anti-Caspase-3, Cleaved Caspase-3, Caspase-8, Cleaved Caspase-8, Caspase-9, Cleaved Caspase-9, PARP, and Cleaved PARP (F). Band intensity was measured by densitometer (G–J). *P < 0.05 and **P < 0.01, the statistically significant difference between MPTP and other groups. The non-adjusted full images for immunoblots are shwon in Fig. S4.

To investigate the role of permeable Tat-PIM2 in the MPTP animal model, we identified the alterations of intracellular antioxidant and apoptosis pathways using Western blotting analysis. Using robust antioxidant markers, such as, SOD1 and catalase, we demonstrated that the antioxidant activity in the Tat-PIM2 treated group was higher compared to the control group (Fig. 5A–C). Moreover, we analyzed the levels of 4-HNE and 8-OHdG to confirm the antioxidant effects of permeable Tat-PIM2 in mice brains. As shown in Fig. 5A, D and 5E, 4-HNE and 8-OHdG conspicuously decreased in the Tat-PIM2 treated group as compared to the MPTP, PIM2, and Tat-peptide treated groups. These results indicate that Tat-PIM2 induces the expression of antioxidant proteins, which effectively suppressed oxidative stress-induced lipid peroxidation and DNA damage in MPTP animal model.

In PD patients, apoptosis of dopaminergic neurons in the SN leads to a dopamine deficit [1,2]. Therefore, we also demonstrated the protective effect of Tat-PIM2 via Caspase signaling for the apoptosis pathway in SN (Fig. 5F–J). Tat-PIM2 increased the levels of full-length Caspases and PARP while the activations of Caspases and PARP dropped. In the PD animal model, MPTP stimulated the activation of apoptosis, but, in contrast with PIM2 and Tat-peptide groups, permeable Tat-PIM2 alleviated Caspase signaling. We thus suggest that Tat-PIM2 could exhibit antioxidant and anti-apoptotic effects on the MPTP animal model.

## 4 Discussion

PD is one of the neurodegenerative diseases and reveals the death of dopaminergic neurons in SN leading to impair dopamine levels [1,2,5]. The cause of dopaminergic neuronal cell death involves in various factors including oxidative stress induced ROS which is particularly recognized to vitally increase and affect the progression of PD [3,4].

PIM2, known as a serine/threonine kinase, modulates the survival and proliferation of cells [[20], [21], [22]] and it has been reported that PIM2 advanced cell survival by controlling growth factor signaling, BAD, NF-κB, and apoptosis signaling [[23], [24], [25]]. However, the precise role of PIM2 on oxidative stress-induced cell death in PD has not yet been studied yet. To investigate the protective role of PIM2 protein in PD, PIM2 was fused with PTD like Tat which can transduce the target protein into cells and tissues and several studies demonstrated that PTD fusion proteins can be useful tool for treatment of neuronal diseases [[29], [30], [31], [32], [33], [34],^36^].

In the present study, we evaluated whether cell permeable Tat-PIM2 fusion protein could prevent ROS induced dopaminergic neuronal cell death *in vitro* and *in vivo*. As shown in results, Tat-PIM2 effectively transduced into the nuclei and cytosol of SH-SY5Y cells and dopaminergic neurons in the SN region of mice brain. Although it is generally known that Tat PTD fusion protein is located in the nucleus of cells [41,42], its intracellular location might be slightly different depending on the target proteins [[43], [44], [45]].

To prepare ROS induced cell and PD mice model, we used MPTP and its metabolite, MPP^+^ in SH-SY5Y cells and mice. MPP^+^ is well known as an intracellular ROS inducer in dopaminergic cells including SH-SY5Y and elevated ROS promotes pathological processes in various neurodegenerative disorders including PD [35,[46], [47], [48]]. It has been reported that PIM2 act as the anti-apoptotic mediator in various apoptosis signaling-related diseases [23,49]. Microenvironment of multiple myeloma was found to enhance PIM2 expression by activating the JAK2/STAT3 pathway and the NF-κB pathway to advance cell survival and cleavage of caspase-3 was strikingly reduced by the overexpression of PIM2 in IL-3 deprivation cells, which demonstrated that PIM2 prevented apoptosis by inhibiting caspase-3 signaling [[22], [23], [24], [25], [26],^50^]. In this study, we performed transduced Tat-PIM2 inhibited the apoptotic caspase signaling and reduced the production of ROS induced by MPP^+^ in SH-SY5Y cells. We confirmed that Tat-PIM2 promoted cell survival by lowering ROS production. It is well known that oxidative stress refers to a condition caused by imbalance in the levels of free radicals and radical scavengers. Oxidative damage leads to neurodegeneration, which is observed mainly in brain disorders and several chronic disorders such as inflammation and diabetes [[51], [52], [53]]. Other reports have shown that polyphenols related to oxidative stress and inflammation signaling pathways including MAPK, NF-κB, apoptosis and PI3K/Akt. Since polyphenols can inhibit the cell signaling pathways related oxidative stress and inflammation, they are suggested that polyphenols can be used in the prevention and treatment of depressive disorder [54]. Also, other reports have demonstrated that diabetes is associated with hyperglycemia which mainly due to oxidative stress and inflammation. *Terminalia catappa* with antioxidant, anti-inflammatory and anti-diabetic activity properties prevents hyperglycemia-induced pathological changes by inhibiting various oxidative stress and inflammatory factors such as apoptosis, oxidative stress and inflammatory factors. Therefore, the authors suggest that *Terminalia catappa* may provide an effective natural therapy for hyperglycemia and prevention of the progression of associated diabetes and diabetic complications [55].

Moreover we investigated the protective effects of Tat-PIM2 in MPTP-induced PD animal model. Tat-PIM2 was injected through intraperitoneal (i.p) and this fusion protein passed through the BBB and transduced into dopaminergic neurons of SN. Transduced Tat-PIM2 showed protective effect and inhibited the apoptosis signaling against cell death in a concentration-dependent manner. Consistent results were obtained in previous studies in which antioxidant cell permeable PTD fusion proteins transduced into neuronal cells and protected against neuronal cell death [32,33,36]. As well as showing the protective effect, Tat-PIM2 elevated the level of antioxidant enzymes such as SOD1 and catalase in PD animal models and ROS induced lipid and DNA oxidation were reduced in mice brain. In summary, Tat-PIM2 has the ability to transduce into dopaminergic neuronal cells across BBB and exhibited anti-apoptotic effect by reducing caspase signaling related to the reduction of ROS. We thus suggest that cell permeable Tat-PIM2 could be a possible candidate for the treatment of PD based on its ability to inhibit cell death.

## 5 Further prospects

Protein transduction technology using PTD is well known for their ability to transduce proteins into cells and tissues including the brain by crossing the BBB. Delivery of therapeutic agents is a key point in the development effective therapeutic agents for the treatment of neuronal diseases because therapeutic agents have difficulty crossing the BBB, which is remains a key obstacle to treatment or the development of effective therapeutic agents. To solve this delivery problem, effective approaches will depend on the development of tools that will allow therapeutic agents to cross the BBB. In this study, we showed the protective effect of Tat-PIM2 against MPP^+^ or MPTP-induced dopaminergic neuronal cell death *in vitro* and *in vivo*. Although further study for the exact molecular mechanisms still need to be explained, we expect that Tat-PIM2 may contribute to the development of effective therapeutic agents for neuronal diseases including PD in the further.

## Author contribution statement

Min Jea Shin, Won Sik Eum: Conceived and designed the experiments; Analyzed and interpreted the data.

Gi Soo Youn, Jung Hwan Park, Hyeon Ji Yeo, Eun Ji Yeo, Hyun Jung Kwon, Eun Jeong Sohn: Performed the experiments.

Lee Re Lee, Na Yeon Kim, Su Yeon Kwon, Su Min Kim: Performed the experiments; Contributed reagents, materials, analysis tools or data.

Hyo Young Jung, Duk-Soo Kim, Sung-Woo Cho, Oh-Shin Kwon: Analyzed and interpreted the data.

Dae Won Kim, Soo Young Choi: Conceived and designed the experiments; Analyzed and interpreted the data; Wrote the paper.

## Funding statement

This work was supported by the Basic Science Research Program through the National Research Foundation of Korea (NRF) funded by the Ministry of Education {NRF2021R1F1A1048079 and 2019R1A6A1A11036849}.

## Data availability statement

Data included in article/supp. material/referenced in article.

## Declaration of competing interest

The authors declare that they have no known competing financial interests or personal relationships that could have appeared to influence the work reported in this paper.

## References

[[1] de Lau L.M., Breteler M.M. (2006). Epidemiology of Parkinson's disease. Lancet Neurol..

[2] Przedborski S. (2005). Pathogenesis of nigral cell death in Parkinson's disease. Parkinsonism Relat. Disorders.

[3] Dauer W., Przedborski S. (2003). Parkinson's disease: mechanisms and models. Neuron.

[4] Zhou C., Huang Y., Przedborski S. (2008). Oxidative stress in Parkinson's disease: a mechanism of pathogenic and therapeutic significance. Ann. N. Y. Acad. Sci..

[5] Kalia L.V., Lang A.E. (2015). Parkinson's disease. Lancet.

[6] Wakabayashi K., Tanji K., Mori F., Takahashi H. (2007). The Lewy body in Parkinson's disease: molecules implicated in the formation and degradation of α-synuclein aggregates. Neuropathology.

[7] Armstrong M.J., Okun M.S. (2020). Diagnosis and treatment of Parkinson Disease: a review. JAMA.

[8] Sveinbjornsdottir S. (2016). The clinical symptoms of Parkinson's disease. J. Neurochem..

[9] Connolly B.S., Lang A.E. (2014). Pharmacological treatment of Parkinson disease: a review. JAMA.

[10] Abou-Sleiman P.M., Muqit M.M.K., Wood N.W. (2006). Expanding insights of mitochondrial dysfunction in Parkinson's disease. Nat. Rev. Neurosci..

[11] Sriram K., Pai K.S., Boyd M.R., Ravindranath V. (1997). Evidence for generation of oxidative stress in brain by MPTP: in vitro and in vivo studies in mice. Brain Res..

[12] Dias V., Junn E., Mouradian M.M. (2013). The role of oxidative stress in Parkinson's disease. J. Parkinsons Dis..

[13] Guo J.D., Zhao X., Li Y., Li G.R., Liu X.L. (2018). Damage to dopaminergic neurons by oxidative stress in Parkinson's disease (Review). Int. J. Mol. Med..

[14] Langston J.W., Ballard P., Tetrud J.W., Irwin I. (1983). Chronic parkinsonism in humans due to a product of meperidine-analog synthesis. Science.

[15] Redza-Dutordoir M., Averill-Bates D.A. (2016). Activation of apoptosis signalling pathways by reactive oxygen species. Biochim. Biophys. Acta.

[16] Perier C., Bove J., Vila M. (2012). Mitochondria and programmed cell death in Parkinson's disease: apoptosis and beyond. Antioxidants Redox Signal..

[17] Baytel D., Shalom S., Madgar I., Weissenberg R., Don J. (1998). The human Pim-2 proto-oncogene and its testicular expression. Biochim. Biophys. Acta.

[18] Alvarado Y., Giles F.J., Swords R.T. (2012). The PIM kinases in hematological cancers. Expert Rev. Hematol..

[19] Ayala G.E., Dai H., Ittmann M., Li R., Powell M., Frolov A., Wheeler T.M., Thompson T.C., Rowley D. (2004). Growth and survival mechanism associated with perineural invasion in prostate cancer. Cancer Res..

[20] Mikkers H., Nawijn M., Allen J., Brouwers C., Verhoeven E., Jonkers J., Berns A. (2004). Mice deficient for all PIM kinases display reduced body size and impaired responses to hematopoietic growth factors. Mol. Cell Biol..

[21] Tahvanainen J., Kylaniemi M.K., Kanduri K., Gupta B., Lahteenmaki H., Kallonen T., Rajavuori A., Rasool O., Koskinen P.J., Rao K.V.S. (2013). Proviral integration site for moloney murine leukemia virus (PIM) kinases promote human T helper 1 cell differentiation. J. Biol. Chem..

[22] Yin G., Li Y., Yang M., Cen X.M., Xie Q.B. (2015). Pim-2/mTORC1 pathway shapes inflammatory capacity in rheumatoid arthritis synovial cells exposed to lipid peroxidations. BioMed Res. Int..

[23] Asano J., Nakano A., Oda A., Amou H., Hiasa M., Takeuchi K., Miki H., Nakamura S., Harada T., Fujii S. (2011). The serine/threonine kinase Pim-2 is a novel anti-apoptotic mediator in myeloma cells. Leukemia.

[24] Keane N.A., Reidy M., Natoni A., Raab M.S., O'Dwyer M. (2015). Targeting the Pim kinases in multiple myeloma. Blood Cancer J..

[25] Kreuz S., Holmes K.B., Tooze R.M., Lefevre P.F. (2015). Loss of PIM2 enhances the anti-proliferative effect of the pan-PIM kinase inhibitor AZD1208 in non-Hodgkin lymphomas. Mol. Cancer.

[26] Xin H., Deng Y., Cao J. (2018). Proviral insertion in murine lymphomas 2 promotes stomach cancer progression by regulating apoptosis via reactive oxygen species-triggered endoplasmic reticulum stress. Biochem. Biophys. Res. Commun..

[27] Kabouridis P.S. (2003). Biological applications of protein transduction technology. Trends Biotechnol..

[28] Pardridge W.M. (2012). Drug transport across the blood-brain barrier. J. Cerebr. Blood Flow Metabol..

[29] Bolton S.J., Jones D.N., Darker J.G., Eggleston D.S., Hunter A.J., Walsh F.S. (2000). Cellular uptake and spread of the cell-permeable peptide penetratin in adult rat brain. Eur. J. Neurosci..

[30] Yeo H.J., Shin M.J., Yeo E.J., Choi Y.J., Kim D.W., Kim D.S., Eum W.S., Choi S.Y. (2019). Tat-CIAPIN1 inhibits hippocampal neuronal cell damage through the MAPK and apoptotic signaling pathways. Free Radic. Biol. Med..

[31] Kim S.J., Shin M.J., Kim D.W., Yeo H.J., Yeo E.J., Choi Y.J., Sohn E.J., Han K.H., Park J., Lee K.W., Park J.K., Cho Y.J., Kim D.S., Eum W.S., Choi S.Y. (2020). Tat-biliverdin reductase A experts a protective role in oxidative stress-induced hippocampal neuronal cell damage by regulating the apoptosis and MAPK signaling. Int. J. Mol. Sci..

[32] Eum W.S., Shin M.J., Lee C.H., Yeo H.J., Yeo E.J., Choi Y.J., Kwon H.J., Kim D.S., Kwon O.S., Lee K.W., Han K.H., Park J., Kim D.W., Choi S.Y. (2019). Neuroprotective effects of Tat-ATOX1 protein against MPP^+^-induced SH-SY5Y cell deaths and in MPTP-induced mouse model of Parkinson's disease. Biochimie.

[33] Choi Y.J., Kim D.W., Shin M.J., Yeo H.J., Yeo E.J., Lee L.R., Song Y., Kim D.S., Han K.H., Park J., Lee K.W., Park J.K., Eum W.S., Choi S.Y. (2021). PEP-1-GLRX1 reduces dopaminergic neuronal cell loss by modulating MAPK and apoptosis signaling in Parkinson's disease. Molecules.

[34] Woo S.J., Shin M.J., Kim D.W., Jo H.S., Yong J.I., Ryu E.J., Cha H.J., Kim S.J., Yeo H.J., Cho S.B., Park J.H., Lee C.H., Yeo E.J., Choi Y.J., Park S., Im S.K., Kim D.S., Kwon O.S., Park J., Eum W.S., Choi S.Y. (2015). Effects of low doses of Tat-PIM2 protein against hippocampal neuronal cell survival. J. Neurol. Sci..

[35] Ito K., Eguchi Y., Imagawa Y., Akai S., Mochizuki H., Tsujimoto Y. (2017). MPP^+^ induces necrostatin-1-and ferrostatin-1-sensitive necrotic death of neuronal SH-SY5Y cells. Cell Death Dis..

[36] Kim M.J., Park M., Kim D.W., Shin M.J., Son O., Jo H.S., Yeo H.J., Bin Cho S., Park J.H., Lee C.H., Kim D.S., Kwon O.S., Kim J., Han K.H., Park J., Eum W.S., Choi S.Y. (2015). Transduced PEP-1-PON1 proteins regulate microglial activation and dopaminergic neuronal death in a Parkinson's disease model. Biomaterials.

[37] Park S.W., Yu K.L., Bae J.H., Kim G.N., Kim H.I., You J.C. (2021). Investigation of the effect of Staufen1 overexpression on the HIV-1 virus production. BMB Rep.

[38] Kim J.K., Cho I.J., Kim E.O., Lee D.G., Jung D.H., Ki S.H., Ku S.K., Kim S.C. (2021). Hemistepsin A inhibits T0901317-induced lipogenesis in the liver. BMB Rep.

[39] Koo B.H., Lee J., Jin Y., Lim H.K., Ryoo S. (2021). Arginase inhibition by rhaponticin increases L-arginine concentration that contributes to Ca^2+^-dependent eNOS activation. BMB Rep.

[40] Park H.J., Kim M.K., Kim Y., Kim H.J., Bae S.K., Bae M.K. (2021). Neuromedin B modulates phosphate-induced vascular calcification. BMB Rep.

[41] Cardarelli F., Serresi M., Bizzarri R., Giacca M., Beltram F. (2007). In vivo study of HIV-1 tat arginine-rich motif unveils its transport properties. Mol. Ther..

[42] Zaro J.L., Vekich J.E., Tran T., Shen W.C. (2009). Nuclear localization of cell-penetrating peptides is dependent on endocytosis rather than cytosolic delivery in CHO cells. Mol. Pharm..

[43] Flinterman M., Farzaneh F., Habib N., Malik F., Gaken J., Tavassoli M. (2009). Delivery of therapeutic proteins as secretable TAT fusion products. Mol. Ther..

[44] Caron N.J., Torrente Y., Camirand G., Bujold M., Chapdelaine P., Leriche K., Bresolin N., Tremblay J.P. (2001). Intracellular delivery of a Tat-eGFP fusion protein into muscle cells. Mol. Ther..

[45] Patel S.G., Sayers E.J., He L., Narayan R., Williams T.L., Mills E.M., Allemann R.K., Luk L.Y.P., Jones A.T., Tsai Y.H. (2019). Cell-penetrating peptide sequence and modification dependent uptake and subcellular distribution of green florescent protein in different cell lines. Sci. Rep..

[46] Xie H.R., Hu L.S., Li G.Y. (2010). SH-SY5Y human neuroblastoma cell line: in vitro cell model of dopaminergic neurons in Parkinson's disease. Chin. Med. J..

[47] Chaturvedi R.K., Beal M.F. (2013). Mitochondrial diseases of the brain. Free Radic. Biol. Med..

[48] Shishido T., Nagano Y., Araki M., Kurashige T., Obayashi H., Nakamura T., Takahashi T., Matsumoto M., Maruyama H. (2019). Synphilin-1 has neuroprotective effects on MPP^+^-induced Parkinson's disease model cells by inhibiting ROS production and apoptosis. Neurosci. Lett..

[49] Zirkin S., Davidovich A., Don J. (2013). The PIM-2 kinase is an essential component of the ultraviolet damage response that acts upstream to E2F-1 and ATM. J. Biol. Chem..

[50] Yan B., Zemskova M., Holder S., Chin V., Kraft A., Koskinen P.J., Lilly M. (2003). The PIM-2 kinase phosphorylates BAD on serine 112 and reverses BAD-induced cell death. J. Biol. Chem..

[51] Lushchak V.I., Duszenko M., Gospodaryov D.V., Garaschuk O. (2021). Oxidative stress and energy metabolism in the brain: midlife as a turning point. Antioxidants.

[52] Behl T., Kaur I., Kotwani A. (2016). Implication of oxidative stress in progression of diabetic retinopathy. Surv. Ophthalmol..

[53] Zhao J., Yan Y., Zhen S., Yu L., Ding J., Tang Q., Liu L., Zhu H., Xie M. (2023). LY294002 alleviates bone cancer pain by reducing mitochondrial dysfunction and the inflammatory response. Int. J. Mol. Med..

[54] Behl T., Rana T., Alotaibi G.H., Shamsuzzaman M., Naqvi M., Sehgal A., Singh S., Sharma N., Almoshari Y., Abdellatif A.A.H., Iqbal M.S., Bhatia S., Al-Harrasi A., Bungau S. (2022). Polyphenols inhibiting MAPK signaling pathway mediated oxidative stress and inflammation in depression. Biomed. Pharmacother..

[55] Behl T., Kotwani A. (2017). Proposed mechanisms of *Terminalia catappa* in hyperglycaemia and associated diabetic complications. J. Pharm. Pharmacol..

