# Supplementary material for: Protective effects of cell permeable Tat-PIM2 protein on oxidative stress induced dopaminergic neuronal cell death
Source: Heliyon. 2023 Apr 29;9(5):e15945. doi: 10.1016/j.heliyon.2023.e15945 (PMC10200856; doi:10.1016/j.heliyon.2023.e15945)

Protective effects of cell permeable Tat-PIM2 protein on oxidative stress induced dopaminergic neuronal cell death

Min Jea Shin[a,1], Won Sik Eum[a,1], Gi Soo Youn[a], Jung Hwan Park[a], Hyeon Ji Yeo[a], Eun Ji Yeo[a], Hyun Jung Kwon[a], Eun Jeong Sohn[a], Lee Re Lee[a], Na Yeon Kim[a], Su Yeon Kwon[a], Su Min Kim[a], Hyo Young Jung[c], Duk-Soo Kim[d], Sung-Woo Cho[e], Oh-Shin Kwon[f], Dae Won Kim[b,*] and Soo Young Choi[a,*]

[a]Department of Biomedical Science and Research Institute of Bioscience and Biotechnology, Hallym University, Chuncheon 24252, Korea. [b]Department of Biochemistry and Molecular Biology, Research Institute of Oral Sciences, College of Dentistry, Gangneung-Wonju National University, Gangneung 25457, Korea. [c]Department of Veterinary Medicine & Institute of Veterinary Science, Chungnam National University, Daejeon, 34134, Korea. [d]Department of Anatomy and BK21 FOUR Project, College of Medicine, Soonchunhyang University, Cheonan-si 31538, Korea. [e]Department of Biochemistry and Molecular Biology, University of Ulsan College of Medicine, Seoul 05505, Korea. [f]School of Life Sciences, College of Natural Sciences Kyungpook National University, Taegu 41566, Korea.

Supplementary figures (Fig. S1-S4)

Fig. S1. (Related to Fig. 1)

Non-adjusted full images of immunoblots in **Fig. 1C** and **1D**.

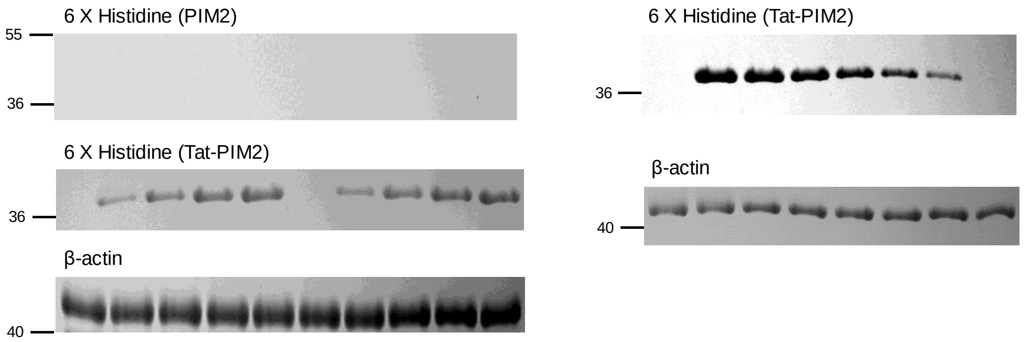

Fig. S2. (Related to Fig. 2)

Non-adjusted full images of immunoblots in **Fig. 2D**.

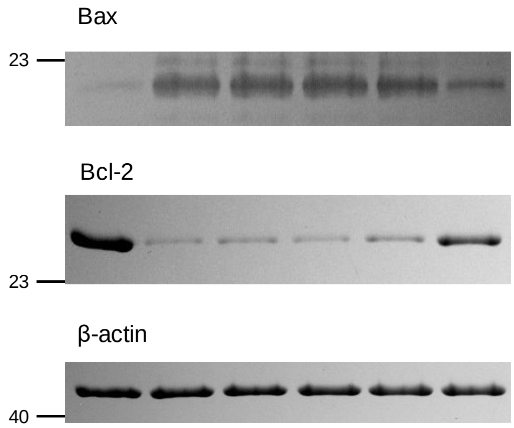

Fig. S3. (Related to Fig. 3)

Non-adjusted full images of immunoblots in **Fig. 3C**.

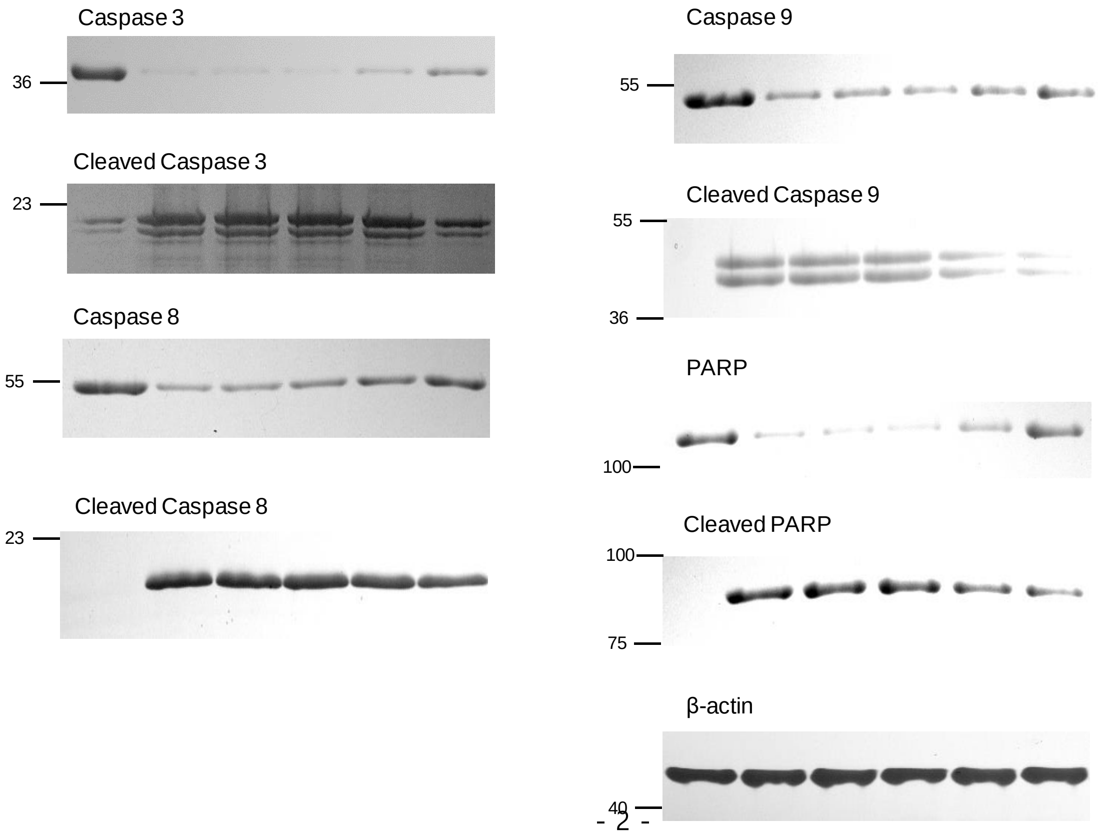

Fig. S4. (Related to Fig. 5)

Non-adjusted full images of immunoblots in **Fig. 5A** and **5F**.

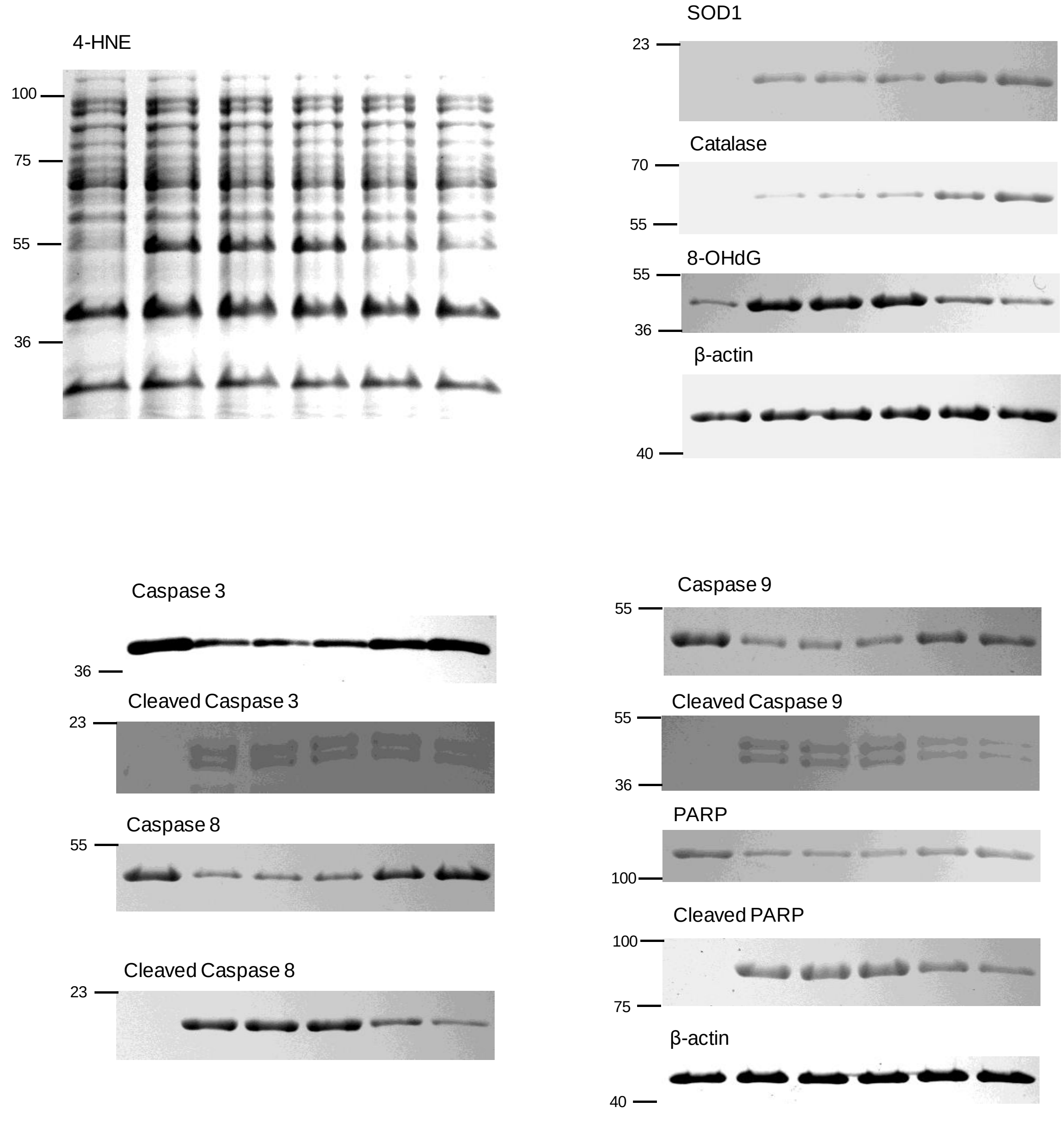

Supplement: Multimedia component 1 [file mmc1.pdf]
